# FAM72A promotes UNG2 degradation and mutagenesis in human cancer cells

**DOI:** 10.1038/s41598-025-07723-x

**Published:** 2025-07-02

**Authors:** Yuqing Feng, Philip Barbulescu, Chetan K. Chana, Melika Shirdarreh, Hong Yang, Lizhen Wu, Sami Mamand, Mohammad Kashem, Amin Zia, Ming Han, Julissa Tsao, Trevor Pugh, David Cescon, David G. Schatz, Frank Sicheri, Alberto Martin, Rossanna C. Pezo

**Affiliations:** 1 Department of Immunology, University of Toronto, Toronto, ON M5S 1A8 Canada; 2 Lunenfeld-Tanenbaum Research Institute, Sinai Health, Toronto, ON M5G 1X5 Canada; 3 Department of Biochemistry, University of Toronto, Toronto, ON M5S 1A8 Canada; 4 Jinan Vocational College of Nursing, Jinan, Shandong China; 5 Department of Molecular Genetics, University of Toronto, Toronto, ON M5S 1A8 Canada; 6 Department of Immunobiology, Yale School of Medicine, New Haven, CT USA; 7 dYcode Bio, Toronto, Canada; 8 Princess Margaret Cancer Centre, Toronto, Canada; 9 Sunnybrook Health Sciences Center, Toronto, ON Canada; 10 Present Address: Department of Biology, York University, Toronto, ON M3J 1P3 Canada

**Keywords:** FAM72, Cancer, Mutation, Uracil DNA glycosylase 2, Cytosine deamination, Base excision repair, Cancer, Immunology, Molecular biology, DNA damage and repair

## Abstract

Genetic lesions drive cancer development and progression, and understanding their origins will reveal the mechanisms of carcinogenesis. We showed that murine FAM72A promotes mutagenic DNA repair during antibody maturation by acting as a substrate adaptor of the CTLH^MKLN1^ E3 ligase to induce the proteasome degradation of Uracil DNA glycosylase 2 (UNG2), a pivotal enzyme of the base excision repair. In humans, the *FAM72* gene has expanded to include four paralogues named *FAM72A-D*. Bioinformatic studies suggested that the human *FAM72* genes are overexpressed in a broad range of cancers. However, the functional roles of FAM72A-D in human biology and cancer are unknown. Here, we show that FAM72 family members are minimally expressed in most healthy tissues except for thymus, and that *FAM72A*, *B* and *D* are overexpressed in primary tumorigenic tissues. Human *FAM72* expression inversely correlates with UNG2 protein level in human cell lines and primary tumorigenic tissues suggesting that human FAM72 promotes UNG2 degradation. However, only FAM72A is able to bind to and induce UNG2 degradation in human cells. Our results suggest that the ability of FAM72A to induce UNG2 degradation contributes to neoplasia in a variety of cancer types by promoting mutagenic repair of genomic dUs.

**Supplementary Information:**

The online version contains supplementary material available at 10.1038/s41598-025-07723-x.

## Introduction

During antigen encounter, activated B cells express Activation-Induced cytidine Deaminase (AID) that deaminates deoxycytidines (dCs) to deoxyuracils (dUs) at the variable and switch regions of *immunoglobulin (Ig)* genes to generate high-affinity and isotype-switched antibodies. dU lesions in DNA are usually recognized and repaired by base excision repair (BER) and mismatch repair pathway enzymes^1,2^. However, instead of being repaired, the dUs are processed by base excision repair (BER) and mismatch repair (MMR) pathways that paradoxically expand the mutations^3–8^. This long-standing puzzle was solved by our work^9^ and that of another^10^ which showed that FAM72A, a protein whose function was previously unknown, promotes mutagenic repair in murine B cells by binding to and causing proteasomal degradation of Uracil DNA glycosylase 2 (UNG2), a component of BER. By degrading UNG2, FAM72A allows AID-produced dUs to persist, leading to repair with mutations^9,11^. FAM72A promotes UNG2 degradation through the E3 C-terminal to LisH (CTLH) ligase complex^12^. Accordingly, FAM72A bridges the interaction between UNG2 and MKLN1, a peripheral subunit of the CTLH complex, and subsequently allows UNG2 to be targeted for polyubiquitination by the MAEA E3 ligase and proteasomal degradation.

The *FAM72* gene has expanded in humans to include four paralogous members called *FAM72A*, *FAM72B*, *FAM72*C, and *FAM72D*^13^. FAM72A is considered as the ancestral member of the family, whereas FAM72B/C/D are human-specific genes not found in the genomes of nonhuman primates, yet are present in archaic *homo* species, including Neanderthals and Denisova^14^. Accumulating studies suggest the *FAM72* gene family is over-expressed in a broad range of cancer types^12,15–28^and elevated *FAM72* expression has been associated with worsened overall survival and disease-free survival outcomes in multiple cancers^12,18–20,25,29^. However, our understanding of the role of FAM72 in oncogenesis is still limited. A few studies have linked the expression of the *FAM72* gene family to cell proliferation, cellular division, and cell cycle progression^17,18,30^. FAM72A has also been proposed to influence the phosphorylation status and cellular function of Protein Phosphatase 2 A (PP2A) substrates including tubulin and Myeloid cell leukemia-1 (Mcl-1) by directly interacting with the PP2A subunit Aα and B56γ^31^. Notably, *FAM72* expression positively correlates with mutation burdens in a broad range of cancer types^12,18,32^suggesting that FAM72A may influence carcinogenesis by promoting mutagenesis.

Characterizing the expression profile of the human FAM72 paralogues serves as a first step towards understanding the functional roles of this gene family in cancer. In this study, we show that the *FAM72* genes are heterogeneously expressed in human cell lines, and *FAM72A*, *B* and *D* are overexpressed in primary human tumors. Knocking out the *FAM72A-D* genes in various human cells lines leads to increased UNG2 expression, and there is an inverse correlation between *FAM72A-D* expression and UNG2 protein levels in breast cancers. We also found that despite a high degree of amino acid sequence similarity between the FAM72 paralogues, only FAM72A is able to bind to and induce UNG2 degradation. As AID and its paralogues from the APOBEC3 (apolipoprotein B mRNA editing enzyme catalytic subunit 3) family of cytidine deaminases are prominent genome mutators^33,34^we propose that FAM72A contributes to mutagenic repair of dUs introduced by the AID/APOBEC3 enzymes, thus playing a role in cancer mutagenesis through a cytosine deamination-dependent mechanism.

## Results

### The FAM72 gene family are expressed at low levels in normal human tissue except the thymus

**Fig. 1:**
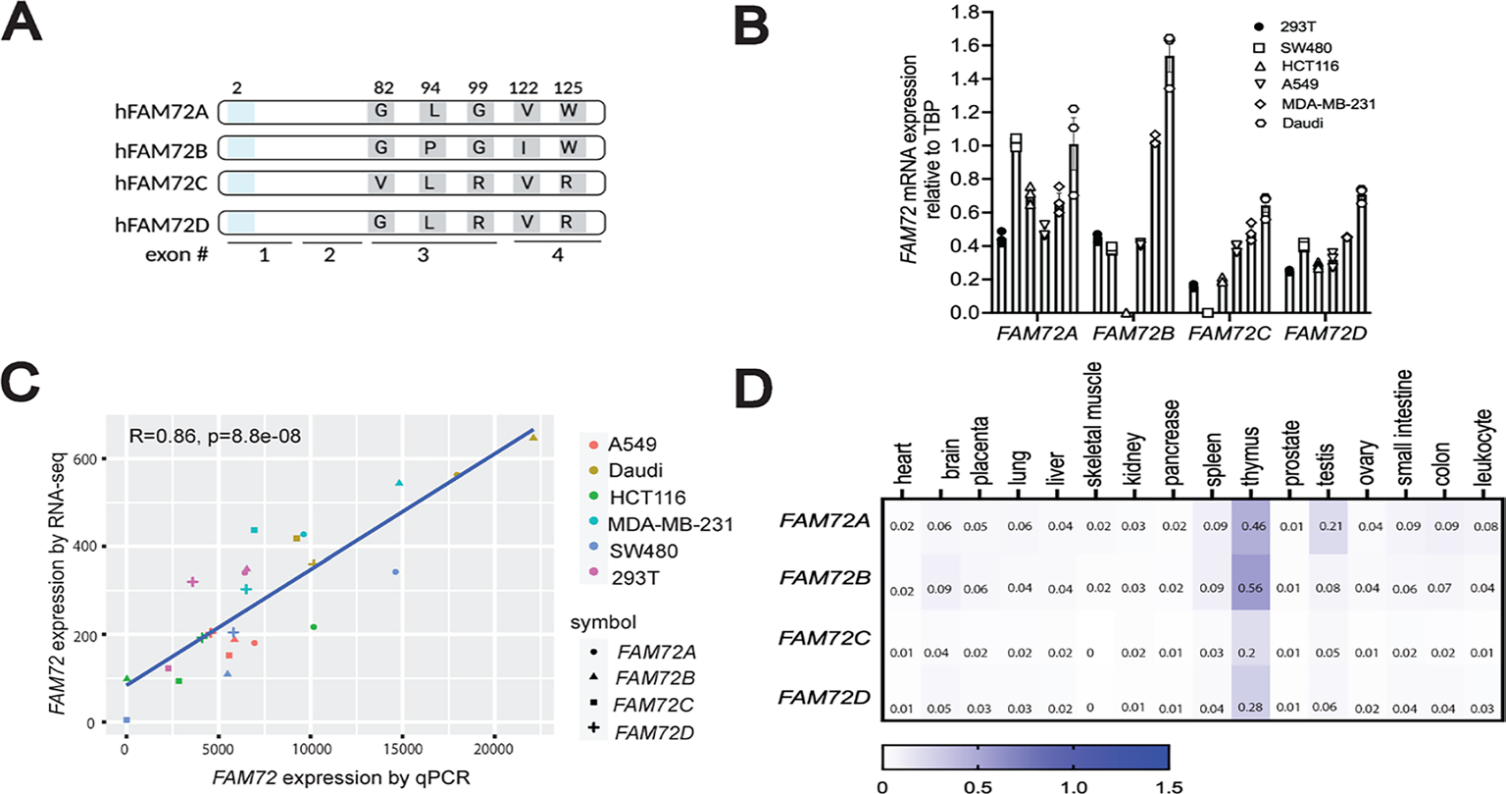
Expression profile of human *FAM72* gene family in cell lines and healthy tissues. (**A**) Schematic of the human FAM72 family members demonstrating that they differ at the amino acid positions 82, 94, 99, 122, and 125. Of note, FAM72 paralogues differ at six nucleotide positions. The substitutions at nucleotide position 6 (amino acid position 2 shown in *blue*) in FAM72C and FAM72D results in silent mutations. Hence, only five out of six nucleotide substitutions result in changes at the protein level. **(B)** Assessment of *FAM72* expression in multiple human cell lines by RT-qPCR. A549: human lung carcinoma; HET293T: human embryonic kidney; MDA-MB-231: human breast adenocarcinoma; HCT116: human colorectal carcinoma; Daudi: human Burkitt’s lymphoma; SW480: human colorectal adenocarcinoma. *FAM72* mRNA expression relative to *TBP* was calculated using the 2^(delta Ct) method. (**C**) Correlation of *FAM72* expression in human cell lines assessed by RNA-seq *versus* qPCR. (**D**) *FAM72* mRNA levels in heathy human tissues as assessed from commercially available multiple tissue cDNA panels. FAM72 transcript levels were compared to housekeeping gene *TBP*. The color scheme provides quantitative information, as the full range of purple (low expression) to red (high expression) is used to as a surrogate measurement of the expression data. Numbers inside each box represent the relative fold change of *FAM72* expression in comparison to TBP calculated using 2^ (delta Ct) method. Three replicates were done for each condition, and the averaged values were shown in the box.

The four human *FAM72* paralogues contain highly homologous 5’ and 3’ untranslated regions and encode proteins that are more than 96% identical and only differ by up to 5 amino acids (Fig. 1A)^13^. Multiple bioinformatic studies showed that the *FAM72* paralogues are overexpressed in human cancers. However, accurate assessment of *FAM72* expression is hampered by the very close nucleotide identity between the four paralogues and data extracted from publicly available RNA-seq databases can be confounded by reads being ascribed to the wrong paralogue, emphasizing the need to carefully assess *FAM72A-D* expression. To confirm and extend results from these previous studies, we developed primers that can specifically measure the expression level of each individual *FAM72* paralogue using qPCR, which differentiate the four *FAM72* paralogues based on mismatches located at the 3’ end of the primer sequence across *FAM72* coding sequences (Fig. S1A). Using linearized control plasmids containing *FAM72* cDNA sequences, the amplification results suggest each qPCR primer pair was highly specific for its target *FAM72* template (Fig. S1B). The primer amplification efficiency was determined using serial dilutions of the same linearized *FAM72* control cDNA templates. We found that the amplification efficiencies for each primer set were high (Fig. S1C).

To further validate the specificity of these primers and reaction, we first measured the expression levels of *FAM72* in a panel of human cell lines derived from different cancer types. Our qPCR data suggests nearly all *FAM72* paralogues are expressed in these cells (Fig. 1B). We then performed RNA-seq on the same cell lines, and found a high degree of correlation for the expression levels of the *FAM72* paralogues using both methods (Fig. 1C**)**, confirming the robustness of the qPCR assay. We next quantified *FAM72* expression in sixteen normal human tissues using commercially available panels of tissue-specific cDNAs pooled from donors. We found *FAM72A-D* expressions were generally low in all tissues, with the exception of human thymus (Fig. 1D). Steady-state *Fam72a* expression was also the highest in murine thymus (Fig. S1D). Collectively, these data show that *FAM72A* and *FAM72B* were expressed 2- to 3-fold higher than *FAM72C* and *FAM72D* in all healthy tissues. *FAM72A* and *FAM72B* are highly expressed in the thymus, suggesting that they may have functional importance in the normal physiology of this organ.

### Colon and breast cancers express high levels of FAM72A, B, and D

**Fig. 2:**
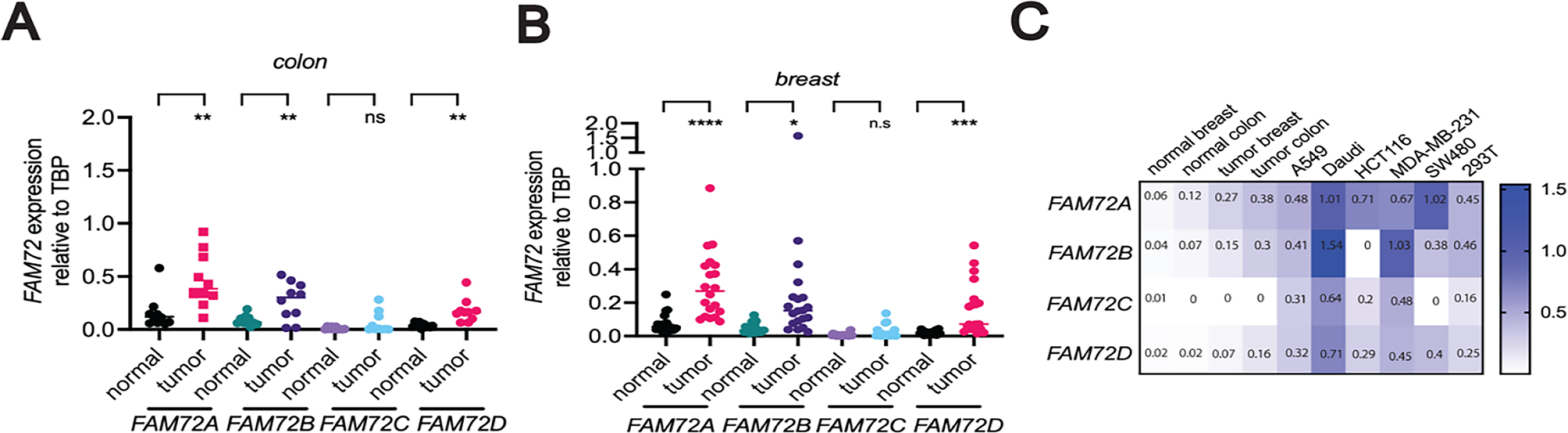
Elevated expression of human *FAM72* genes in primary cancers. (**A**) *FAM72* mRNA expression level in primary colon cancer tissue specimen paired with normal flanking tissue obtained from Ontario Institute for Cancer Research (OICR) as assessed by RT-qPCR using FAM72 paralogue-specific primers. The relative FAM72 transcript expression was compared to *TBP* using 2^(delta Ct) method. (**B**) Same as (**A**), except *FAM72* mRNA expression levels in primary breast cancer tissue specimen are shown. (**C**) Expression of *FAM72* genes in normal and cancer tissues in comparison to human cell lines as shown in Fig. 1B. Purple (low expression) to red (high expression) is used to as a surrogate measurement of expression data. Two-tailed unpaired Student’s t-test was used for statistically analysis. *, *P* < 0.05; **, *P* < 0.01; ***, *P* < 0.001 ****; *P* < 0.0001.

To determine whether primary human cancers express *FAM72* paralogues, we quantified *FAM72* paralogue levels using RNA isolated from paired cancer and flanking control tissues in 10 randomly chosen colon tumors and 20 breast tumor specimens (Table S2). Our data show that the expression levels of all *FAM72* paralogues, with the exception of *FAM72C*, were increased in both colon and breast cancer tissues relative to the normal flanking tissues (Fig. 2A, B, Fig. S2). However, the expression of *FAM72A-D* was generally higher in cell lines than in primary tumors (Fig. 2C). Because the promoter region sequences are nearly identical between the four FAM72 paralogues^30,32^we hypothesized that the *FAM72* genes are co-regulated at the mRNA transcript level. Indeed, we found that the expression levels of *FAM72A-D* are positively correlated with each other (Fig. S3A, B), supporting this notion. In conclusion, by using a paralogue specific qPCR assay, we confirm that *FAM72A*,* B*, and *D* are overexpressed in colon and breast cancers, supporting previous studies that relied on analyzing publicly available databases.

### FAM72 mRNA expression inversely correlates with UNG2 protein expression

**Fig. 3:**
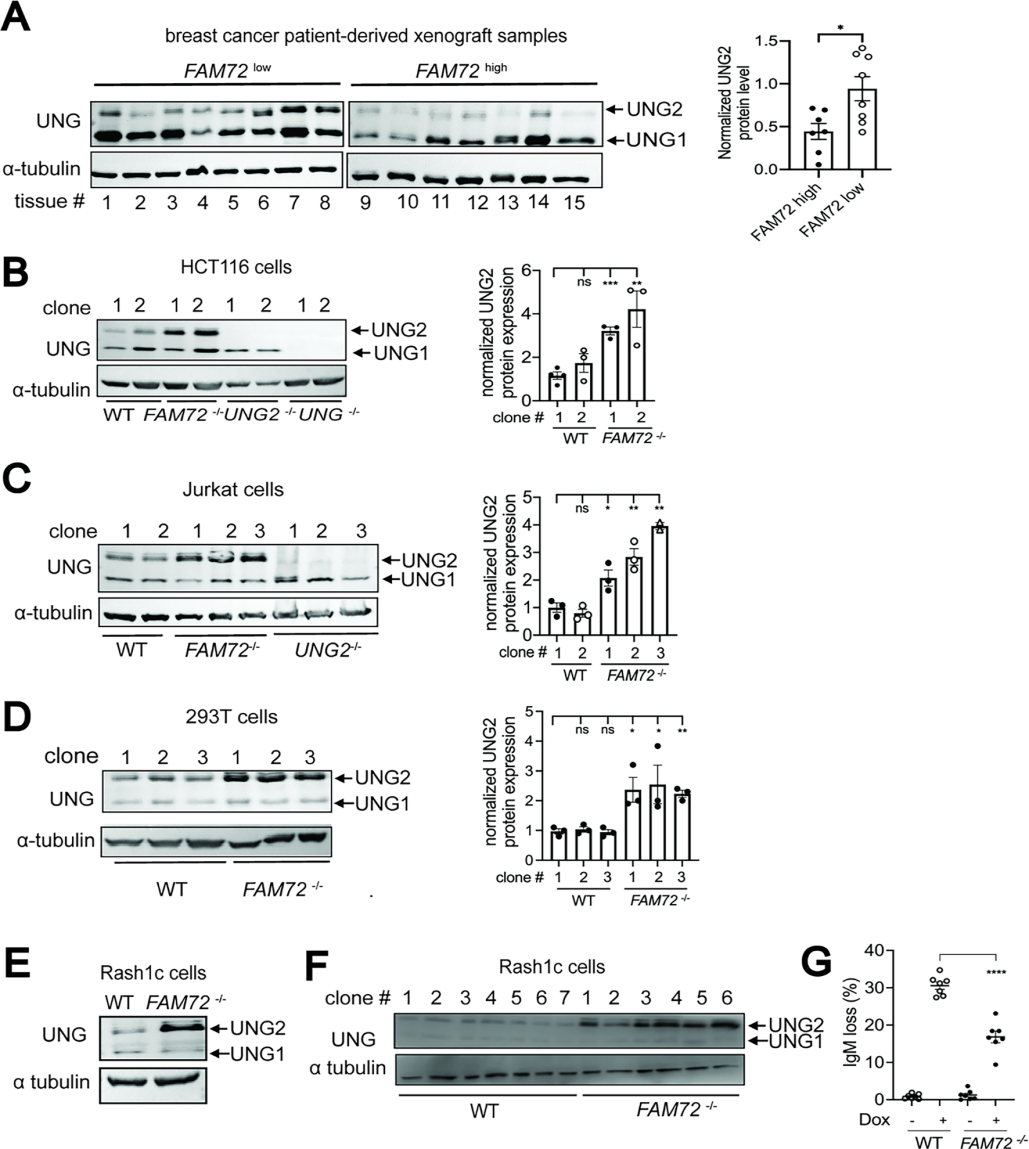
FAM72 expression promotes UNG2 degradation and mutagenesis. (**A**) Western blotting for UNG protein in 15 human xenograft breast cancer tissues that express either high or low level of *FAM72*. Graph on the right shows the expression of UNG2 protein relative to a-tubulin. (**B**) Western blotting of whole cell lysates to assess UNG level in different clones of WT and *FAM72*^−/−^ Cas9-HCT116 cells. Alpha-tubulin was used as a loading control for all immunoblots. Densitometric analysis shown on right was carried out on 3 independent Western blots. (**C**) Same as B, except that UNG expression was analyzed in WT and FAM72-deficient Jurkat cells. (**D**) Same as B, except that UNG expression was analyzed in WT and FAM72-deficient 293 T cells. (**E**) Western blotting of whole cell lysates to assess UNG levels in WT and *FAM72*^−/−^ Cas9-expressing RASH1c cells. (**F)** Western blotting of whole cell lysates to assess UNG level in seven independent subclones derived from WT and *FAM72*^−/−^ RASH1c cells. (**G**) Quantification of IgM loss in WT and *FAM72*^−/−^ RASH1c cells treated with or without doxycycline (dox) for 4 days. Two-tailed unpaired Student’s t-test was used for statistically analysis. *, *P* < 0.05; **, *P* < 0.01; ***, *P* < 0.001 ****; *P* < 0.0001.

FAM72A induces the proteolytic degradation of UNG2 in murine cells^9,10,12^. To determine whether a similar function is preserved in human cells, we examined UNG2 protein levels in human xenograft breast cancer tissues that have been stratified based on *FAM72* transcript levels (Fig. S4A). Indeed, we found that FAM72 levels inversely correlated with UNG2 levels (Fig. 3A), suggesting that FAM72 degrades UNG2 degradation in tumorigenic tissues. To confirm that FAM72 degrades UNG2 in human cells, we knocked out *FAM72* in various human cell lines through lentivirus-mediated CRISPR genome editing by designing a guide RNA that targets a region in *FAM72* exon 2 that is common in all four paralogues (Fig. S4B). We edited the genomes of the human colon cancer cell lines HCT116, and two *FAM72*-deficient HCT116 clones were validated by genotyping and qPCR (Fig. S4C, D). Our results suggest that knocking out *FAM72* led to a ~ 3-fold increase in UNG2 protein level in HCT116 (Fig. 3B). Similar results were obtained in *FAM72-*deficient Jurkat cells (Fig. 3C and Fig. S4E) and 293 T cell lines **(**Fig. 3D and Fig. S4E).

We also examined the effects of knocking out the *FAM72* genes in RASH1c cells, which is a Cas9 expressing variant of the human Ramos B cell line that can be used to rapidly assess SHM frequencies^35^ (Fig. S4F). *FAM72-*deficient RASH1c cells were generated by CRISPR/Cas9 editing (Fig. S4E). FAM72 deficiency led to increased UNG2 protein (Fig. 3E), and this increase was also observed in subclones derived from this population (Fig. 3F). Importantly, doxycycline treatment of these cells, which induces AID-expression, led to reduced IgM loss in *FAM72-*deficient RASH1c cells compared to controls (Fig. 3G). As IgM loss is a well-established surrogate measurement for mutagenesis during SHM in RASH1c cells^35^these data indicate that FAM72 supports AID-induced mutations during SHM. In conclusion, these results show that FAM72 expression inversely correlated with UNG2 protein in human cells suggesting that FAM72 has the same function as in murine cells.

### FAM72A, but not FAM72B-D, is responsible for promoting UNG2 degradation

**Fig. 4:**
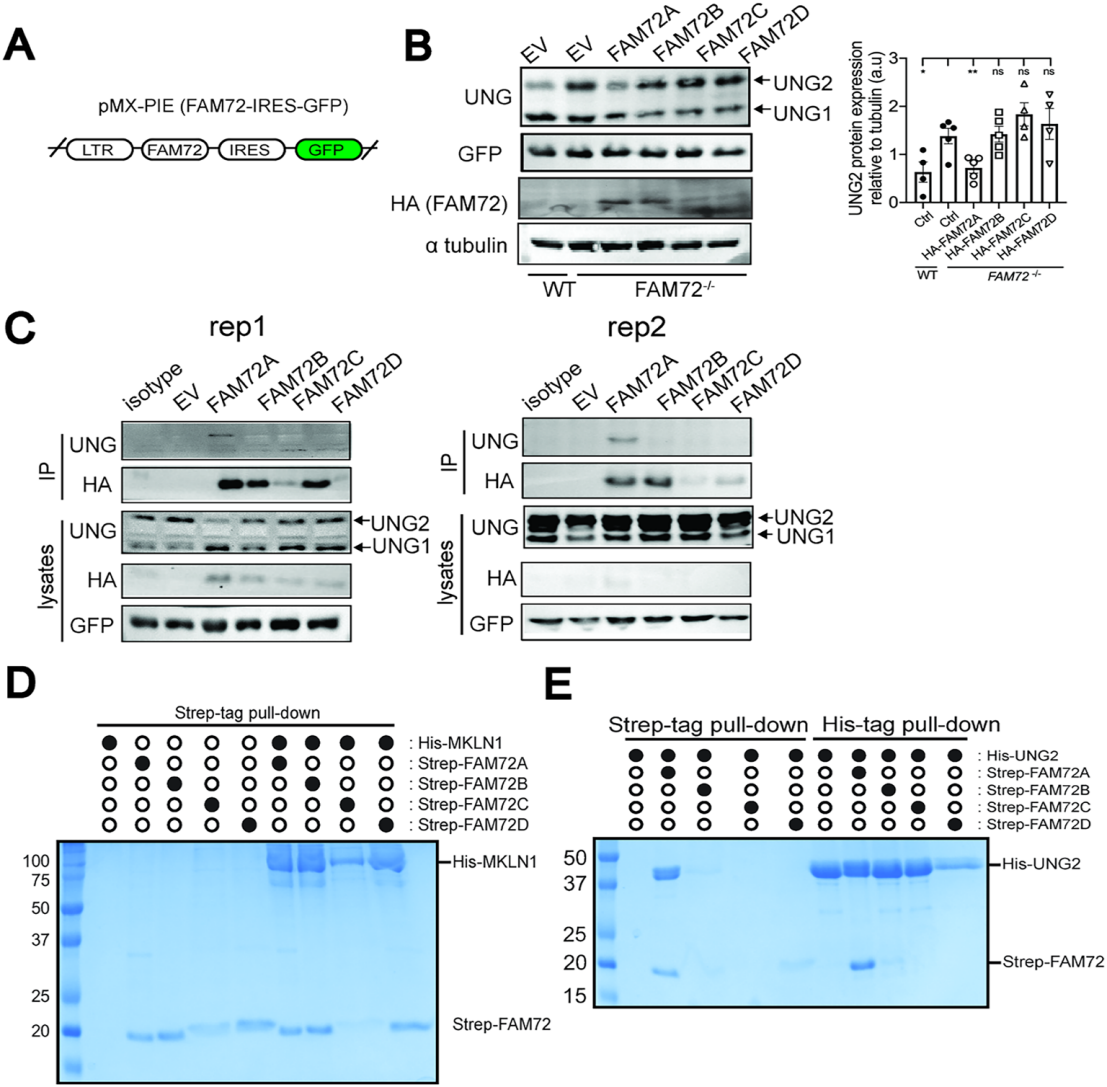
FAM72A, but not FAM72B-D, binds to and induces UNG2 degradation in human cells. (**A**) Schematic showing the pMX-Pie-HA-FAM72A-GFP expression vector that was used to transduce *FAM72*^−/−^ HCT116 cells. (**B**) Western blotting of whole cell lysates to examine HA-FAM72 and UNG level in *FAM72*^−/−^ HCT116 cells expressing pMX-PIE empty vector (EV), or pMX-PIE HA-tagged FAM72A/B/C/D. Cells were sorted based on GFP expression after viral transduction, and GFP level was assessed based on Western blot. Alpha-tubulin was used as a loading control. Densitometric analysis was done with 3 independent experiments. Two-tailed unpaired Student’s t-test was used for statistically analysis. (**C**) Immunoprecipitation of HA-FAM72 in MG132-treated HCT116 cell transduced with the indicated constructs. Two independent experiments were shown. (**D**) Strep-Tag II pull-down from *Sf9* lysates expressing the indicated His_6x_-tagged MKLN1 and Strep-Tag II FAM72A/B/C/D proteins, followed by visualization by Coomassie-stained SDS-PAGE. **(E)** His_6x_-tag and Strep-Tag II pull-downs from *Sf9* lysates expressing the indicated Strep-Tag II FAM72A/B/C/D and His_6x_-tagged UNG2 proteins, followed by visualization by Coomassie-stained SDS-PAGE. Data is presented as mean ± s.d of a minimum of 3 biological replicates. ns, not significant; *, *P* < 0.05; **, *P* < 0.01.

To determine which specific FAM72 paralogue is able to induce UNG2 degradation in human cells, we ectopically expressed HA-tagged FAM72A, B, C, or D in *FAM72*-deficient HCT116 clone 1 using a retroviral vector encoding *FAM72*s and *GFP* that are separated by an Internal Ribosomal Entry Site (IRES) (Fig. 4A). Transduced cells were sorted for GFP expression to normalize for variation in transduction efficiency. Synonymous mutations were introduced at the protospacer adjacent motif (PAM) and gRNA binding site to minimize editing of the FAM72 cDNA by Cas9 that is stably expressed in the *FAM72*-deficient HCT116 cells (Fig. S5A). *FAM72* paralogue gene expression was confirmed using qPCR (Fig. S5B). Our results suggest cells that express FAM72A, but not FAM72B-D, were able to decrease UNG2 protein level (Fig. 4B).

To determine whether the variance in UNG2 degradation caused by the FAM72 paralogues is due to different UNG2 binding capacities, we compared the abilities of HA-tagged FAM72 paralogues to co-immunoprecipitate endogenous UNG2. *FAM72*^*-/-*^ HCT116 cells expressing HA-tagged FAM72A, B, C, or D were treated with MG132 to block proteasome degradation of FAM72 to better visualize FAM72-UNG2 interactions. We found that HA-FAM72 expression levels are low in regular lysates even after MG132 treatment; however, detection is improved under immunoprecipitation conditions which enrich for proteins. Nevertheless, our data indicates that only FAM72A is able to interact with UNG2 (Fig. 4C). To further examine the interaction of the FAM72 paralogues with UNG2, we co-expressed Strep-tagged FAM72 with His-tagged UNG2 or MKLN1 in *Sf9* insect cells to assess their interactions using an in vitro pull-down assay. Interacting with MKLN1, a peripheral member of the CTLH E3 ligase complex, is required for FAM72A to induce the proteolytic degradation of UNG2 in murine cells^12^. We found that all human FAM72 paralogues are able to bind MLKN1 **(**Fig. 4D**)**. However, only FAM72A is able to robustly bind to UNG2 (Fig. 4E). Collectively, these data show that FAM72A is the only paralogue able to bind to and cause the degradation of UNG2 protein.

## Discussion

Despite the recognized importance of FAM72A in murine cells and its fundamental role in supporting mutagenic repair during antibody maturation^9–12^this function needs to be confirmed in human cells. In addition, further work is needed to assess whether FAM72A contributes to any additional functional activities, especially in cancer. While bioinformatic studies suggest that the human *FAM72* gene family members are highly expressed in a broad range of cancer types, there has been limited experimental validation of the cell line and tissue expression profile of the human FAM72 family members. In this study, we show that *FAM72* paralogues were minimally expressed in nearly all healthy human tissues, and that *FAM72A*, *B*, and *D* were upregulated in primary tumorigenic tissues and tumor cell lines. It is unclear why FAM72 expression is elevated in most cancers (this study and^12^). Our data suggests that *FAM72* paralogue expressions are co-regulated at the transcript level (Fig. S3). Future work is required to identify promoters and transcription factors required for FAM72 paralogue gene expression, and mechanisms that contribute to *FAM72* upregulation in cancer.

The precise functions of FAM72A, B, and D in cancer still requires further investigation. Our study here suggests that among the four FAM72 paralogues, only FAM72A can downregulate UNG2, and may explain the increased mutagenesis in cancers that overexpress FAM72A^12^. Since FAM72A promotes AID-induced mutagenesis^9,10^we hypothesize that FAM72A will support APOBEC3A, B-mediated mutagenesis in non-B cells. These enzymes are homologues of AID and linked to cancer development^34,36^. FAM72A may also contribute to genome instability in cancer development because low levels of UNG2 as observed in FAM72A ^high^ cancers (Fig. 3A) can lead to increased level of unprocessed genomic dUs, which is a source of replication stress that can interfere with replication fork progression^37^. FAM72A and other FAM72 paralogues such as FAM72B and FAM72D may also contribute to carcinogenesis by modulating cell proliferation and cell cycle progression^17,31^. Nevertheless, our study suggests that interfering with FAM72A-UNG2 interaction through small molecular inhibitors might represent a potential avenue to treat cancers with high FAM27A expression.

## Methods

### Cell culture

A549 (human lung carcinoma, gift from Dr. Daniel Schramek, Lunenfeld-Tanenbaum Research Institute), HET293T (human embryonic kidney; OpenBiosystems, Cat# HCL4517), MDA-MB-231 (human breast adenocarcinoma; gift from Dr. Daniel Schramek, Lunenfeld-Tanenbaum Research Institute), HCT116 (human colorectal carcinoma; A. Martin laboratory stock, Daudi (human Burkitt’s lymphoma; A. Martin laboratory stock), and SW480 (human colorectal adenocarcinoma; A. Martin laboratory stock) were cultured under DMEM supplemented with 10% FBS and penicillin-streptomycin. Jurkat (gift from Dr. Tania Watts, University of Toronto) and RASH1c (gift from Dr. David Schatz, Yale School of Medicine) cells were cultured under RPMI1640 supplemented with 10% FBS, 50 µM of β-Mercaptoethanol, and penicillin-streptomycin. All cells were cultured at 37 °C with 5% CO2.

### Mice

C57BL/6 Wild-type mice (Charles River Laboratory, 6 to10 week-old) were bred in our Animal Vivarium facility at the University of Toronto. The experimental procedures were performed in accordance with the guidance and regulations approved by the Animal Care Committee of University of Toronto (Protocol number: 20011472). Our animal experiments comply with the ARRIVE guidelines.

### Plasmids

The coding sequence for human FAM72A was ordered from IDT and cloned into mammalian expression vector pcDNA3.1 and retroviral vector pMX-PIE using BamHI and NotI sites, and FLAG-HA-pcDNA3.1 (N-terminal FLAG-HA tagged, Addgene, plasmid #52535) using XbaI and HindIII sites. Site-directed mutagenesis was used to construct FAM72B, FAM72C, and FAM72D using FAM72A sequence as a template. Guide RNA (gRNA) targeting human *FAM72*, *UNG2*, and *UNG* were cloned into lentiCRISPRv2 (Addgene, plasmid #52961). Primers were obtained from Invitrogen or IDT and sequences are listed in Table S1. All plasmid constructs were verified by sequencing at The Centre for Applied Genomics (Toronto, ON).

### Lentiviral and retroviral transduction

To generate lentiviruses, HEK 293 T cells were seeded in DMEM with 10% FBS and penicillin-streptomycin, and 18 h later, were transfected with lentiviral packing plasmids pMD2.G (Addgene, plasmid #12259) and psPAX (Addgene, plasmid #12260), along with lentiviral plasmids containing Cas9 and gRNAs of interest in the presence of PEI. Lentivirus or retrovirus-containing supernatants were harvested 48 h after transfection, and filtered through a 0.45-µm filter before being used to infect target cells. Lentiviruses were concentrated using ultracentrifugation.

### CRISPR–Cas9-mediated gene editing in human cancer cell lines

To knock out genes of interest in HCT116, 293 T, and Jurkat cells, the lentiviruses encoding Cas9 and gRNA against *FAM72*,* UNG*2, or *UNG* were used to transduce these cells. At 24 h post-transduction, transduced cells were selected with 8 µg/mL of puromycin for 7 days, and subcloned by limiting dilution. *FAM72* was knocked out in RASH1c cells using the same method as published^35^. Individual knockout clones were validated by sanger sequencing, quantitative PCR, and/or Western blot.

### Genotyping FAM72 edited alleles

The ~ 1 kb region spanning guide RNA binding site in edited *FAM72* loci was PCR amplified from 293 T, Jurkat, RASH1c, and HCT116 cells using Platinum SuperFi II DNA polymerase (Thermofisher Scientific, catalog no. 12361010) with primers listed in Table S1. Purified *FAM72* PCR products were cloned into Zeroblunt PCR vector (Thermofisher Scientific, catalog no. K275020) and transformed into competent TOP10 bacteria. The plasmids yielded from individual bacterial clones were sequenced at The Centre for Applied Genomics (Toronto, ON).

### Measuring UNG2 protein level in patient-derived xenograft samples

Patient-derived xenograft (PDXs) were generated by implanting tumor tissues from human primary or metastatic breast cancer lesions in immunocompromised female mice. Following engraftment and passaging, PDX samples were collected and snap frozen. Frozen tumor fragments were lysed in 1X Laemmli buffer (125 mM Tris-HCl pH 6.8, 4% SDS and 20% glycerol) containing 0.5uL benzonase/mL buffer following dissociation with 1 metal bead/tube (Qiagen # 69989) and shaking on a TissueLyzer for 4 min at 20hz and room temperature incubation for 20 min. Informed consent was obtained from all participants and/or their legal guardians where breast tumor material was obtained. The experimental procedures were performed in accordance with the guidance and regulations and under Research Ethics Board-approved protocols at the Princess Margaret Cancer Centre (Protocol number: UHN #15–9481). All procedures involving human participants were conducted in accordance with the Declaration of Helsinki.

### Western blotting

Proteins were detected by chemiluminescence after the nitrocellulose membranes were probed with antibodies. The antibodies used were anti-human UNG (Invitrogen, catalog no. MA5-25686, clone OTI2C12), alpha-tubulin (Abcam, catalog no. ab4074), anti-HA (Cell Signaling, clone C29F4, catalog no. 3724 S), anti-GFP (Invitrogen, catalog no. A-11122). Blots were developed with Invitrogen iBright 1500 imaging system (Thermofisher). Densitometry analysis was performed using ImageJ.

### Immunoprecipitation

HCT116 *(FAM72*-deficient cells expressing empty vector control or HA-tagged FAM72A/B/C/D) were seeded in 15 cm dish until they reach near 75% confluency. Cells were then treated with 20 µM of MG132 (Sigma, cat# 474790-10MG) for 6 h before harvesting. After that, cells were trypsinized, pelleted, washed twice in cold PBS, weighed, and snap-frozen at −80 °C. On day of immunoprecipitation, samples were thawed on ice, resuspended in non-ionic IP lysis buffer (40 mM Tris-HCL pH 7.5, 150 mM NaCl, 1 mM EDTA pH 8.0, 10 mM NaF, 1% NP-40 substitute, 50 µM NEM, cOmplete mini protease inhibitor), and lysed for 2 h at 4 °C. Rabbit IgG or Rabbit-HA antibodies were added to washed protein G beads and rolled at 5 rpm for 3 h at 4 °C to allow binding. Beads were washed twice with IP wash buffer, added to precleared cell lysate, and rolled overnight at 5 rpm. Whole cell lysates from samples were collected following preclearing. On day two, beads were washed 4x in IP wash buffer (40 mM Tris-HCL pH 7.5, 150 mM NaCl, 1 mM EDTA pH 8.0, 10 mM NaF, 0.5% NP-40 substitute, 5% glycerol), resuspended in 6x Laemmli buffer, boiled, and used for Western blotting with 0.2 µM nitrocellulose membranes (BioRad). Membranes were probed for HA, GFP and UNG expression. VeriBlot-HRP for IP Detection Reagent (Abcam, ab131366, 1:200) was used as the secondary antibody, the reaction was developed with Pierce ECL substrate (ThermoFisher), and membranes were visualized through the iBright Imaging System (ThermoFisher).

### Pull-down from Sf9 cell lysates

Sf9 insect cells were co-transfected with baculovirus for expression of the indicated proteins as discussed previously^12^. Cells were harvested and lysed by sonication in 20mM HEPES pH 7.5, 500mM NaCl, 250 µM TCEP, 20mM imidazole (Buffer A) and 0.05% Brij-25 supplemented with 1x cOmplete, EDTAfree protease inhibitor cocktail tablet (Sigma-Aldrich) and 1mM PMSF at 4 °C. Cleared lysates were split into two aliquots and each aliquot incubated for 30 min with either Ni^2+^-NTA (EMD Millipore) or Strep-Tactin XT 4 Flow (IBA Lifesciences) resin. The resin was washed 5 times with Buffer A, resuspended in Laemmli buffer and visualized by SDS-PAGE and Coomassie staining.

### RT-qPCR

RT-qPCR was performed as discussed previously^9^. Briefly, total RNA extraction was performed using either Trizol (Invitrogen) or Purelink RNA mini kit (Invitrogen, cat# 12183018 A) according to the manufacture’s protocol. Unless otherwise noted, one microgram of RNA has been used to synthesize complementary DNA (cDNA) using Maxima H Minus reverse transcriptase (ThermoFisher) and oligo-dT primers. The cDNA samples were synthesized using PowerUp SYBR Green Master Mix (Applied Biosystems) according to the manufactures protocols. The PCR product was amplified using a Bio-rad CFX 384 touch real-time PCR detection system. *TBP* were used as house-keeping genes for RT-qPCR, and FAM72 expression levels were standardized by the 2-$\:\varDelta\:$Ct method. The primer amplification specificity for each *Fam72* gene was confirmed by testing it against a linearized plasmid DNA containing cDNA copy of *FAM72*.

### RNA-seq

RNA extracted from various cell lines were sequenced at the Princess Margaret Genomic Centre, Toronto. Briefly, RNA libraries were prepared from 100ng of total RNA using the Illumina Stranded Total RNA Prep Ligation with Ribo-Zero Plus. Libraries were sequenced with pair-end 100 cycles V3 using Illumina Novaseq6000 to a target depth of 40 million reads per sample. FASTQs were aligned to the hg38 human reference genome using STAR 2.7.2b [Dobin,2013] aligner with default settings. Expression levels of all transcripts were quantified using RSEM 1.3.0 [Li,2011] with the GENCODE transcript reference version 31. RNA data quality metrics were collected using RNAseQC v1.1.8 [DeLuca,2012]. RSEM quantified normalized read counts per transcript were used as input for differential expression analyses.

### Correlation of FAM72 paralog expression assessed by RNA sequencing versus RT-qPCR

RSEM quantified gene expression raw counts were normalized using DESeq2 [Love,2014] normalization method and compared to RT-qPCR gene expression values for *FAM72A-D* across 6 cell lines (A549, Daudi, HCT116, MDA-MB-231, SW480 and 293 T). Pearson correlation and p-value were calculated based on the mean expression of two RNA sequencing replicates and three RT-qPCR replicates.

### IgM loss assay

RASH1c cells were seeded at 1 × 10^5^ cells in 24 well in the presence of 200 ng/mL doxycycline, and harvested on day 4-post doxycycline induction. Cells were stained with IgM-PE antibody (Southern Biotech, no. 9020-09) and assayed for IgM loss by flow cytometry.

### Statistics and survival analysis

All statistical analyses were performed in the R Statistical Computing Environment v4.0.1 (R Foundation for Statistical Computing, Vienna, Austria. URL https://www.R-project.org/). Cox proportional hazards regression modeling was done using the survival R package. KM plots were made using survminer R package. Other plots were made using ggplot2 R package. All analyses were performed in GraphPad Prism software. Otherwise noted, two-tailed unpaired Student’s *t*-test was used.

## Electronic supplementary material

Below is the link to the electronic supplementary material.

Supplementary Material 1

Supplementary Material 2

Supplementary Material 3

## Data Availability

The data that support the findings of this study are available from the corresponding author, Y.F., upon request. The raw RNA-seq data are available in the Gene expression omnibus (GEO) under accession number GSE299474.
