# Supplementary material for: FAM72A promotes UNG2 degradation and mutagenesis in human cancer cells
Source: Sci Rep. 2025 Jul 2;15:23467. doi: 10.1038/s41598-025-07723-x (PMC12223117; doi:10.1038/s41598-025-07723-x)

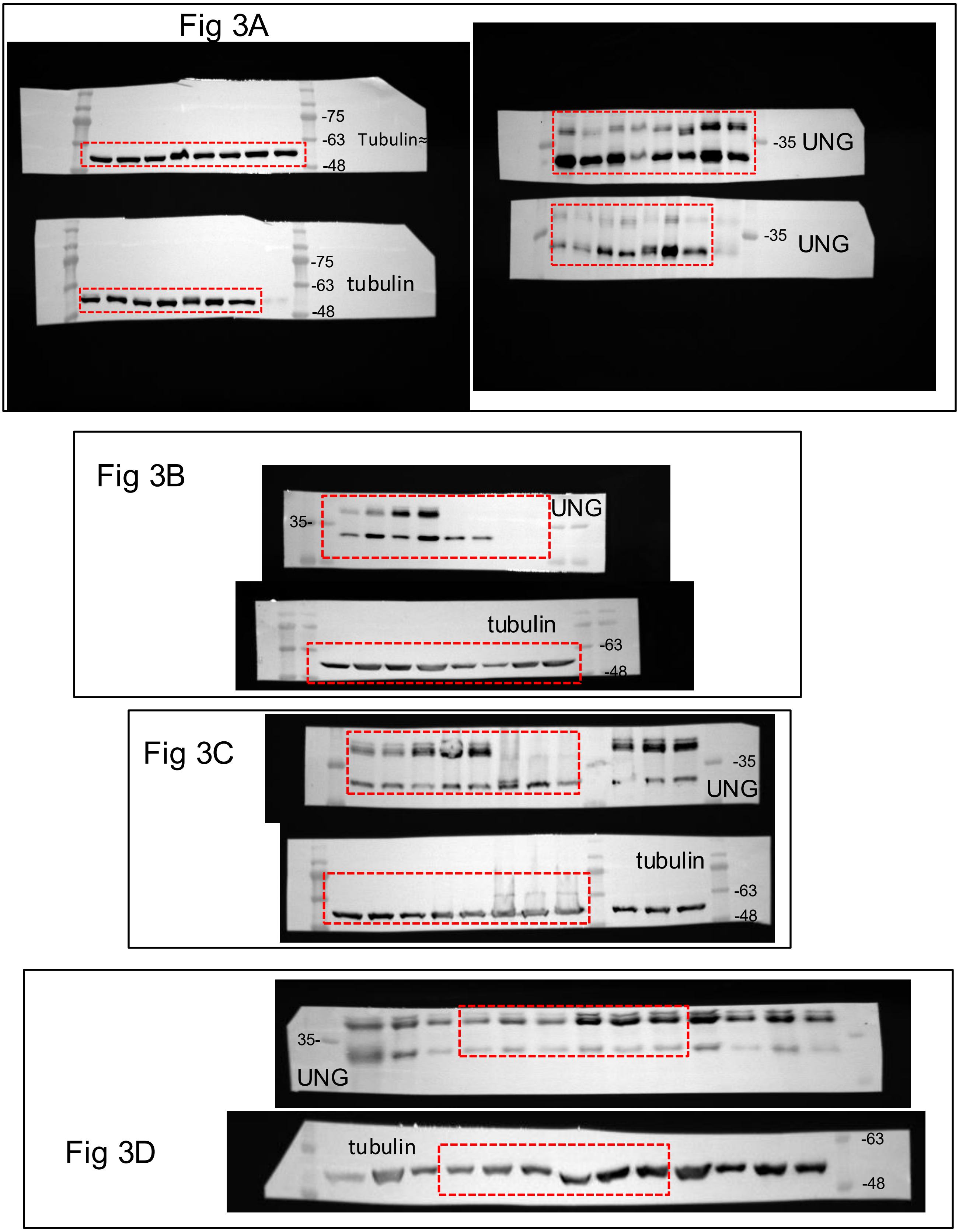

Supplementary info Figure 1: uncropped gel images. Images used to construct the indicated figure panels are listed.

Fig 3E

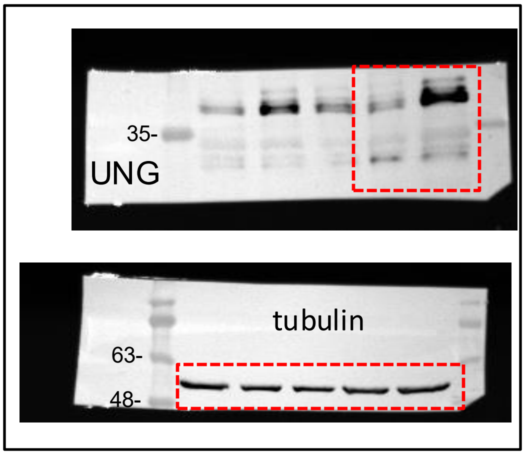

Fig 3F

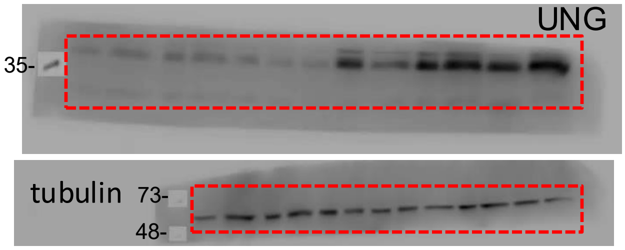

Fig 4B

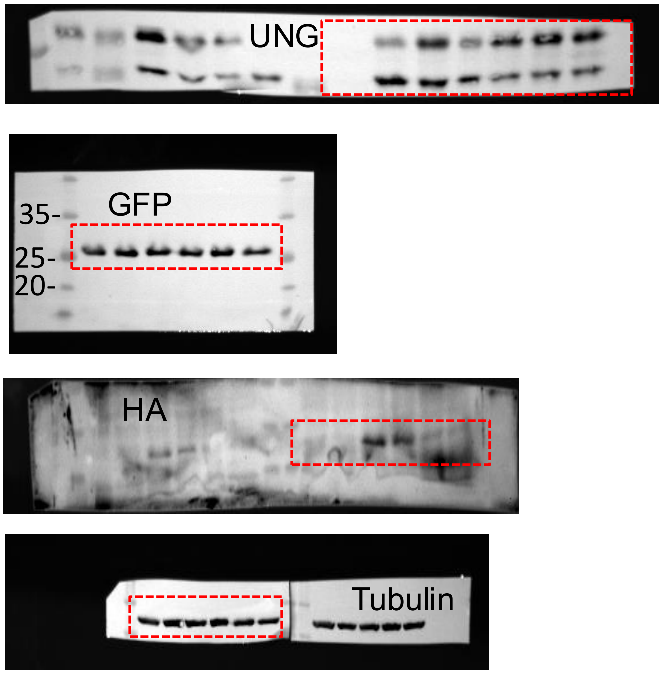

Fig 4C; rep 1

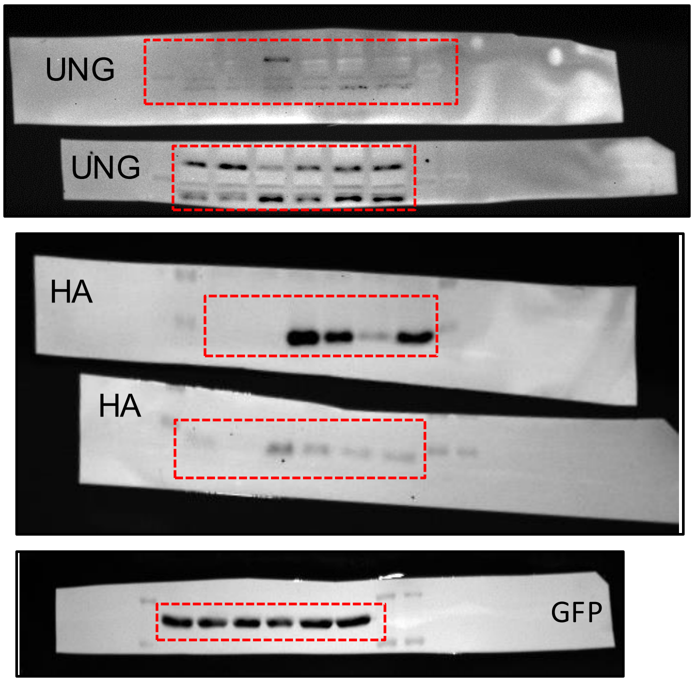

Fig 4C; rep 2

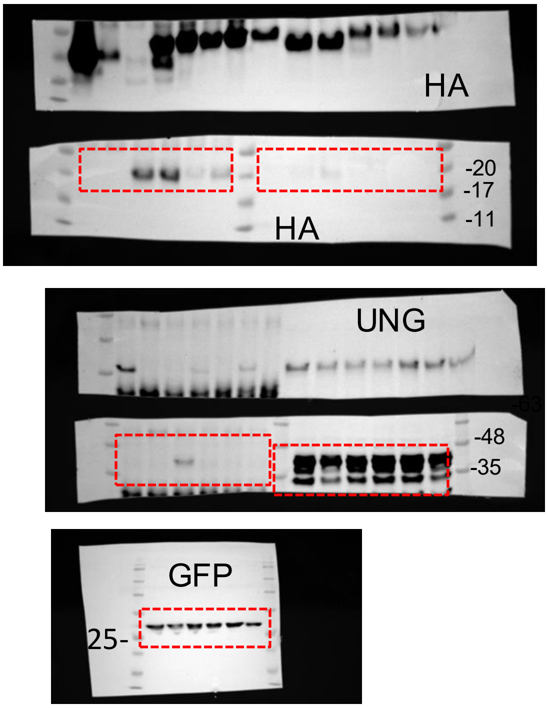

Supplement: Supplementary file 1 — Supplementary Material 1 [file 41598_2025_7723_MOESM1_ESM.pdf]
