# Supplementary material for: FAM72A promotes UNG2 degradation and mutagenesis in human cancer cells
Source: Sci Rep. 2025 Jul 2;15:23467. doi: 10.1038/s41598-025-07723-x (PMC12223117; doi:10.1038/s41598-025-07723-x)
Supplement: Supplementary file 2 — Supplementary Material 2 [file 41598_2025_7723_MOESM2_ESM.pdf]

**Table S1**: primers and oligos used in this study

| name | sequence (5' to 3') | application |
|---|---|---|
| hFAM72A_SNP_F | TCTTCTTTCCTGCAACAACG | FAM72A-sepecific qPCR primer |
| hFAM72A_SNP_R | AAGTTGCCCCAAAGTAGGAC | FAM72A-sepecific qPCR primer |
| hFAM72B_SNP_F | TCTTCCTTCCTGCAACAACG | FAM72B-sepecific qPCR primer |
| hFAM72B_SNP_R | AAGTTGCCCCAAAGTAGGAT | FAM72B-sepecific qPCR primer |
| hFAM72C_SNP_ F1 | AAAATGTGGGAACATTGTAGT | FAM72C-sepecific qPCR primer |
| hFAM72C_SNP_ R2 | ATCTCTGGCAAGTTGCCCCG | FAM72C-sepecific qPCR primer |
| hFAM72D_SNP_F2 | AAAATGTGGGAACATTGTAGG | FAM72D-sepecific qPCR primer |
| hFAM72C_SNP_ R1 | TGAAACATCCAGAAGTGTCT | FAM72D-sepecific qPCR primer |
| hFam72a_gRNA_e2.1_W | CACCGAATCTGCAAATGTAAACTGA | gRNA targeting FAM72 in 293T, Jurkat, and HCT116 |
| hFam72a_gRNA_e2.1_C | AAACTCAGTTTACATTTGCAGATTC | gRNA targeting FAM72 in 293T, Jurkat, and HCT116 |
| hUNG_sgRNA_e2_w | caccgGCGGCCCGCAACGTGCCCGT | gRNA targeting UNG |
| hUNG_sgRNA_e2_c | aaacACGGGCACGTTGCGGGCCGCc | gRNA targeting UNG |
| hUNG2 sgRNA e1.3_w | caccGGGCGTGTCGCTTCCTGGCG | gRNA targeting UNG2 |
| hUNG2 sgRNA e1.3_c | aaacCGCCAGGAAGCGACACGCCC | gRNA targeting UNG2 |
| hFam72_genotyping F1 | TTCTACCCTTCTTTGTAGGGG | genotyping Fam72 ko clone in 293T, Jurkat, and HCT116 |
| tFam72a-1Rv | CCCCCTAACCTACATTGCCTT | genotyping Fam72 ko clone in 293T, Jurkat, and HCT116 |
| hUNG_PCR_e2_F | CTGGGACCTGTTCCACAAAT | genotyping UNG ko clone |
| hUNG_PCR_e2_R1 | GCTCAAGCCAGGTTTCATTC | Genotyping UNG ko clone |
| hUNG2 e1_TIDE Forward 3 | CAGGATGATAATTGCTGACCGC | Genotyping UNG2 ko clone |
| hUNG2 e1_TIDE Reverse 3 | CCCCTCTCCCCTCTGATTGG | Genotyping UNG2 ko clone |
| hTBP qPCR_F | cccatgactcccatgacc | RT-qPCR |
| hTBP qPCR_R | tttacaaccaagattcactgtgg | RT-qPCR |

| | | |
|---|---|---|
| hGAPDH qPCR_F | TCCACCACCCTGTTGCTGTAG | RT-qPCR |
| hGAPDH qPCR_R | GACCACAGTCCATGCCATCACT | RT-qPCR |
| hFam72a_pcDNA_Bamh1_F2 | ttttggatccgccaccATGTCTACCAACATTTGTAGT | cloning |
| hFam72a_NotI_R2 | ttttgcggccgcTTATCTAATACACTCCTCTGC | cloning |
| hFam72_gRNA_e1_W | caccgAGCATGACTTACTTGGTAGG | gRNA targeting FAM72 in RASH1c |
| hFam72_gRNA_e1_c | aaacCCTACCAAGTAAGTCATGCTc | gRNA targeting FAM72 in RASH1c |
| Genotyping_hFAM72_e1F2 | GAC AGC AGA AAG GGA AAG CC | genotyping Fam72 ko clone in RASH1c |
| Genotyping_hFAM72_e1R2 | ACG GAT CTG ATT TAG GAT GCC | genotyping Fam72 ko clone in RASH1c |
| Fam72a_qPCR_F1 | TTTCAAAGACCGATGCGTATCC | qPCR |
| Fam72a_qPCR_R1 | CTATGTCAGTATCAGCCAGCAAA | qPCR |
| mouse HPRT qPCR F | cccagcgtcgtgattagc | qPCR |
| mouse HPRT qPCR F | ggaataaacacttttttccaaat | qPCR |

**Table S2: Clinic report for breast and colon cancer specimens.**

| Sample I.D | Grade | Site of disease | ER status | PR status | HER2 status | Tumor size (cm) | Pathological T | Pathological N |
|---|---|---|---|---|---|---|---|---|
| 1A/B-breast | X | PRIM | Positive NOS | Positive NOS | Negative | 3.5 | T2 | NX |
| 1C/D-breast | III (high-poorly differentiated) | PRIM | Positive NOS | Positive NOS | Negative | 3.1 | T2 | N0, NOS |
| 2E/F-breast | III (high-poorly differentiated) | NODES,PRIM | Negative | Negative | Positive, NOS (FISH) | 6 | TX | NX |
| 2G/H-breast | III (high-poorly differentiated) | NODES,PRIM | Positive NOS | Negative | Positive, NOS (FISH) | 6 | T3 | N2a |
| 3A/B-breast | II (intermediate, moderately | NODES, PRIM | Positive NOS | Positive NOS | Negative | 3.6 | T2 | N1a |

| | | | | | | | | |
|---|---|---|---|---|---|---|---|---|
| | differentiated) | | | | | | | |
| 3C/D-breast | III (high-poorly differentiated) | NODES, PRIM | Negative | Negative | Positive, NOS (FISH) | 7.3 | TX | NX |
| 3E/F-breast | I (low-well differentiated) | NODES, PRIM | Positive NOS | Positive NOS | Negative | 3.6 | T2 | N2a |
| 3G/H-breast | III (high-poorly differentiated) | PRIM | Weakly staining | Negative | Positive, NOS (IHC) | 3.2 | T2 | N0, NOS |
| 4A/B-breast | II (intermediate, moderately differentiated) | PRIM | Positive NOS | Positive NOS | Negative | 3.5 | T2 | N0, NOS |
| 4C/D-breast | III (high-poorly differentiated) | NODES, PRIM | Negative | Negative | Positive NOS | 2.5 | T2 | N3a |
| 4E/F-breast | III (high-poorly differentiated) | PRIM | Negative | Negative | Negative | 2.6 | T2 | N0, NOS |
| 5A/B-breast | III (high-poorly differentiated) | NODES, PRIM | Negative | Negative | Negative | 3.3 | T2 | N1a |
| 6G/H-breast | I (low,well differentiated) | PRIM | Positive NOS | Positive NOS | Negative | 6 | T3 | NX |
| 7A/B-breast | X-unknown | NODES,PRIM | Positive NOS | Positive NOS | Intermediate | 2.4 | N/A | N/A |
| 7C/D-breast | II (intermediate, moderately differentiated) | NODES,PRIM | Positive NOS | Positive NOS | Negative | 6.5 | T3 | N1a |
| 7E/F-breast | II (intermediate, moderately differentiated) | NODES,PRIM | Positive NOS | Positive NOS | Negative | 6.5 | T3 | N2a |
| 7G/H-breast | III (high-poorly differentiated) | NODES,PRIM | Positive NOS | Negative | Positive NOS (IHC) | 13 | T3 | N2a |
| 8A/B-breast | I (low,well differentiated) | NODES,PRIM | Positive NOS | Positive NOS | Pending | 1.8 | T1c | N2a |

| | | | | | | | | |
|---|---|---|---|---|---|---|---|---|
| 8C/D-breast | II (intermediate, moderately differentiated) | PRIM | Positive NOS | Negative | Negative | 4 | T2 | N0, NOS |
| 8E/F-breast | III (high-poorly differentiated) | NODES, PRIM | Weakly staining | Negative | Positive, NOS (FISH) | 4 | T2 | N2a |
| 1C/D-colon | II | PRIM | | | | 3.5 | T3 | N0 |
| 1E/F-colon | II | NODES,PRIM | | | | 7 | T4 | N0 |
| 1G/H-colon | II | PRIM | | | | 4.5 | T3 | N0 |
| 2A/B-colon | IV | NODES,PRIM,LIVER | | | | 6 | T3 | N2 |
| 5C/D-colon | II | NODES,PRIM | | | | 4 | T3 | N1 |
| 5E/F-colon | II | PRIM | | | | 7 | T3 | N0 |
| 5G/H-colon | II | PRIM | | | | 5 | T2 | N0 |
| 6A/B-colon | IV | NODES,PRIM | | | | 7 | T4 | N1 |
| 6C/D-colon | II | NODES,PRIM | | | | 4 | T3 | N1 |
| 6E/F-colon | II | PRIM | | | | 6 | T3 | N0 |

ER: estrogen receptor; PR: progesterone receptor; HER2: human epidermal growth factor receptor 2; NOS, not otherwise specified; pathological T: indicate the primary tumor status determined pathologically at the time of diagnosis and prior to any treatment; TX: Main tumor cannot be measured; T1-4: size/extent of the tumor. Pathological N: indicate the extent of regional lymph nodes involvement determined pathologically at the time of diagnosis and prior to any treatment; NX: cancer in nearby lymph nodes cannot be measured; N0: no cancer in nearby lymph nodes; N1-3: number of lymph nodes that contain cancer.
