# Supplementary material for: FAM72A promotes UNG2 degradation and mutagenesis in human cancer cells
Source: Sci Rep. 2025 Jul 2;15:23467. doi: 10.1038/s41598-025-07723-x (PMC12223117; doi:10.1038/s41598-025-07723-x)
Supplement: Supplementary file 3 — Supplementary Material 3 [file 41598_2025_7723_MOESM3_ESM.docx]

**
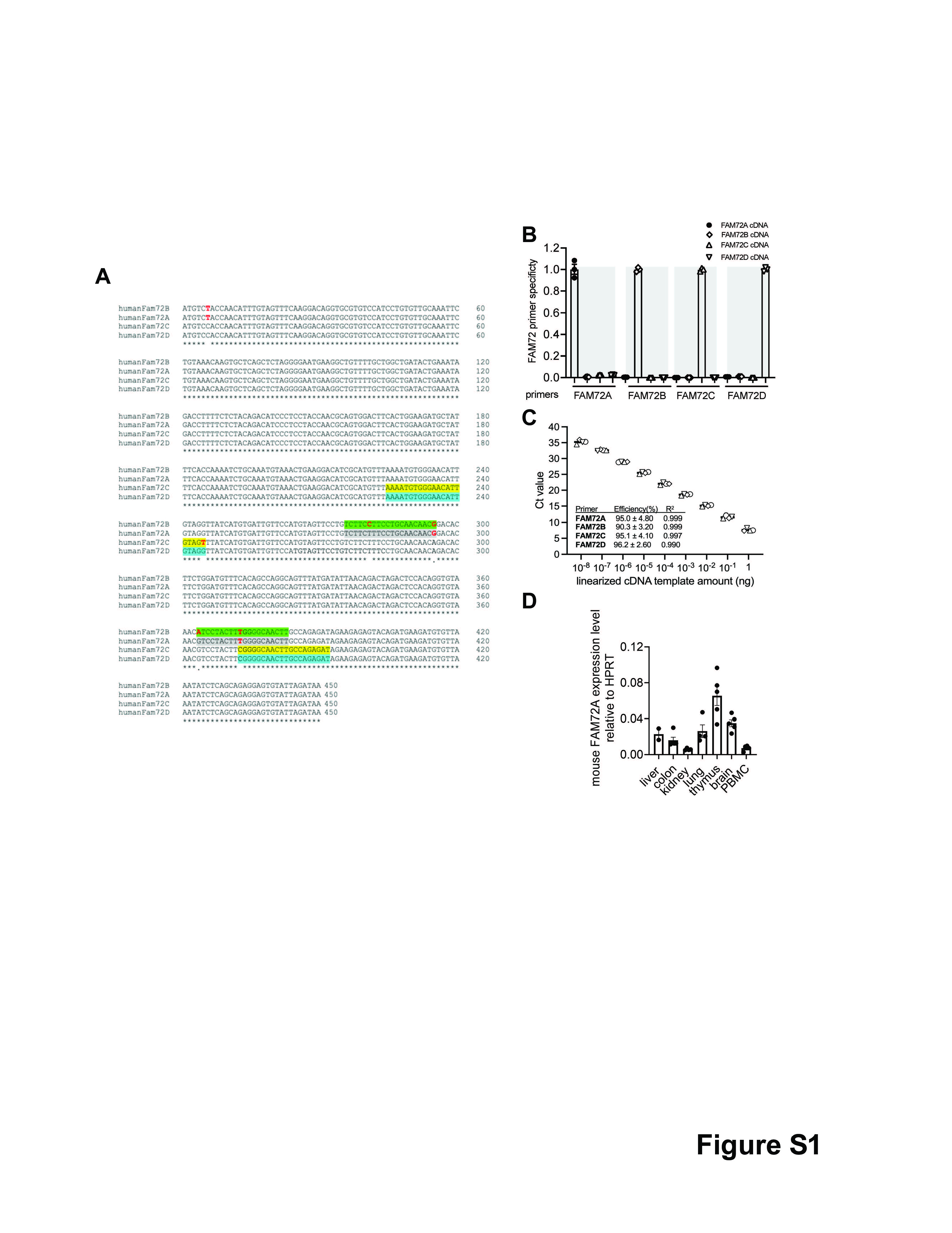
Figure S1. Development of qPCR primers that specifically amplify each FAM72 paralogue.** (**A**) Nucleotide sequence alignment of *FAM72* gene family open reading frames. The primer pairs that lead to the amplification of FAM72A (shown in *grey*), FAM72B (shown in *green*), FAM72C (shown in *yellow*), and FAM72D (shown in *blue*) are color coded. Mismatches located at the 3’ end of the primers are bolded in *red*. (**B**) Primer specificity test. Primer specificity was tested by running primer pair against 10^-4^ ng of each of the PvuI-linearized *FAM72A-D* cDNA templates. (**C**) Primer amplification efficiency test. Serial dilution of the indicated linearized FAM72 cDNA templates was used in qPCR to measure efficiencies. The mean and standard deviation of three independent experiments are shown. (**D**) *Fam72a* expression levels in various organs isolated from C57BL/6J mice. HPRT was used as a housekeeping gene. Freshly harvested tissues were homogenized using the routine grinding method over 70 µm cell strainer, washed, and cell pellets were stored in Trizol until ready for RT-qPCR. Each dot represents an individual mouse.

**

Figure S2**: ***FAM72* paralogue expression levels in breast and colon cancer patients.** (**A**) *FAM72A, B, C, D* mRNA expression levels in 20 paired individual normal and breast cancer patient tissues are shown relative. Each point represents the mean RNA level of three qPCR reactions, presented relative to the housekeeping gene *TBP*. **(B**) Same as A, except that expression is shown for 10 paired normal and colon cancer patient tissues.

**
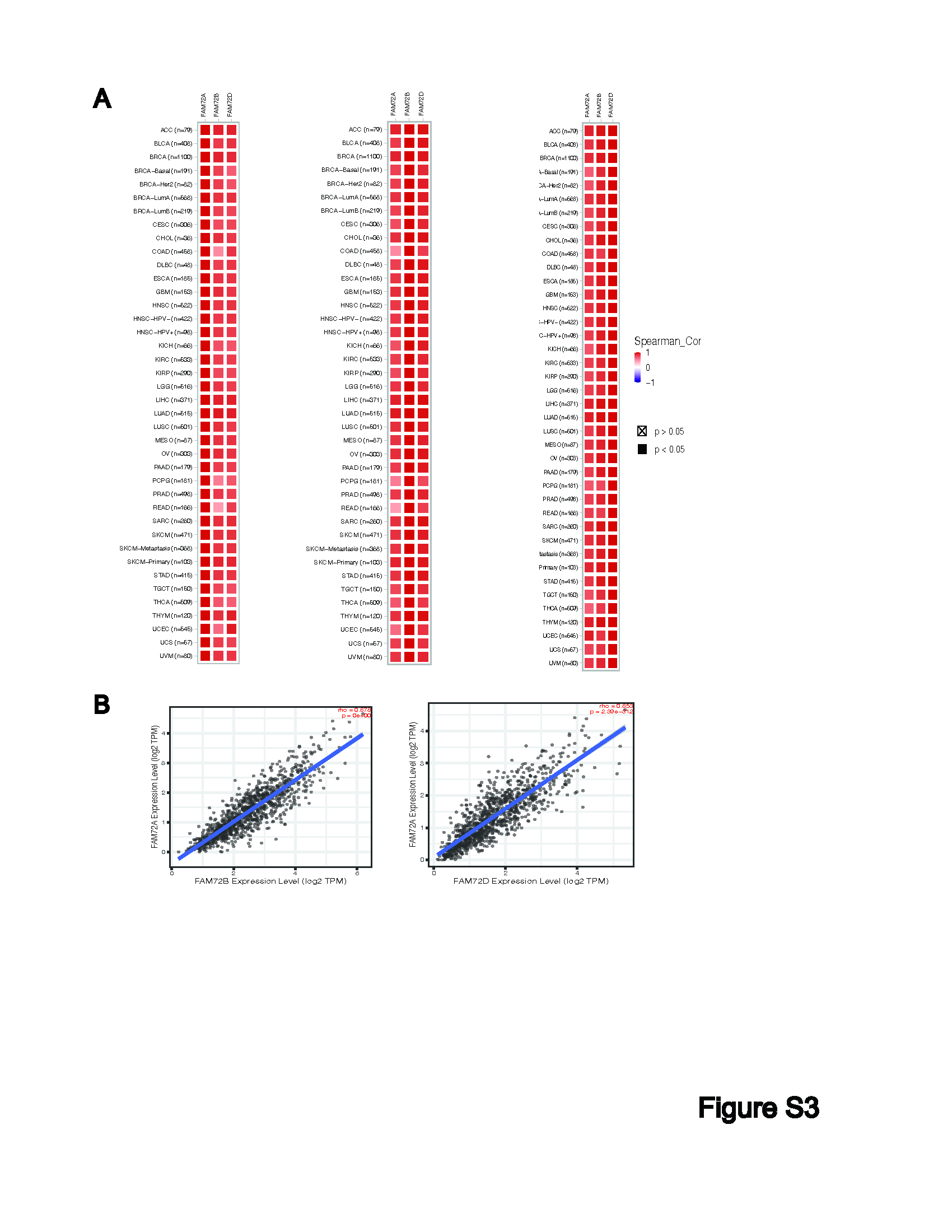
Figure S3. Co-regulated expression of FAM72A, B, and D**. (**A**) The expression of FAM72A, B, and D in RNA-seq datasets available at The Cancer Genome Atlas (TCGA). (**B**) Correlation plot of log2(TPM+ 0.001) for *FAM72A* and *FAM72B,* as well as *FAM72A* and *FAM72D* expression, in breast invasive carcinoma (BRCA).

**

Figure S4. Generation of *FAM72*-deficient human cell lines using Cas9-mediated genome editing.** (**A**) RNAseq of human xenograft breast cancer tissues. Shown are the average of *FAM72A-D* expression in each tumor relative to *UNG* mRNA, and subsequently grouped into FAM72^high^ and FAM72^low^ for Western blots shown in **Fig. 3A**. (**B**) Sequence of guide RNA that targets a region in exon 2 common to all 4 FAM72 paralogues was designed and shown in red. Protospacer Adjacent Motif (PAM) site (AGG) was highlighted in bold. (**C**) The genotypes of edited FAM72 alleles in HCT116 cells. CRISPR target region in FAM72 exon 2 was PCR amplified using primers that amplify all 4 FA M72 paralogues and subcloned into a cloning vector for sequencing. Underlined sequence denotes gRNA target site, with the wildtype amino acid sequence indicated at the bottom. (**D**) Quantification of *FAM72* mRNA relative to TBP in wild-type and *FAM72*^-/-^ HCT116 clones by qPCR. Two-tailed unpaired Student’s t-test was used for statistically analysis. **(E**) Genotyping analysis of FAM72-deficient clones in 293T and Jurkat cells. Displayed are percentages of the edited population detected by TIDE algorithm. (**F**) The genotypes of edited FAM72 alleles in RASH1c cells. CRISPR target region in FAM72 exon 1 was PCR amplified using primers that amplify all 4 FAM72 paralogues and subcloned into a cloning vector for sequencing. Underlined sequence denotes gRNA target site, with the wildtype amino acid sequence indicated at the bottom. *, P < 0.05; **, P < 0.01; ***, P < 0.001 ****; *P < 0.0001*.

**
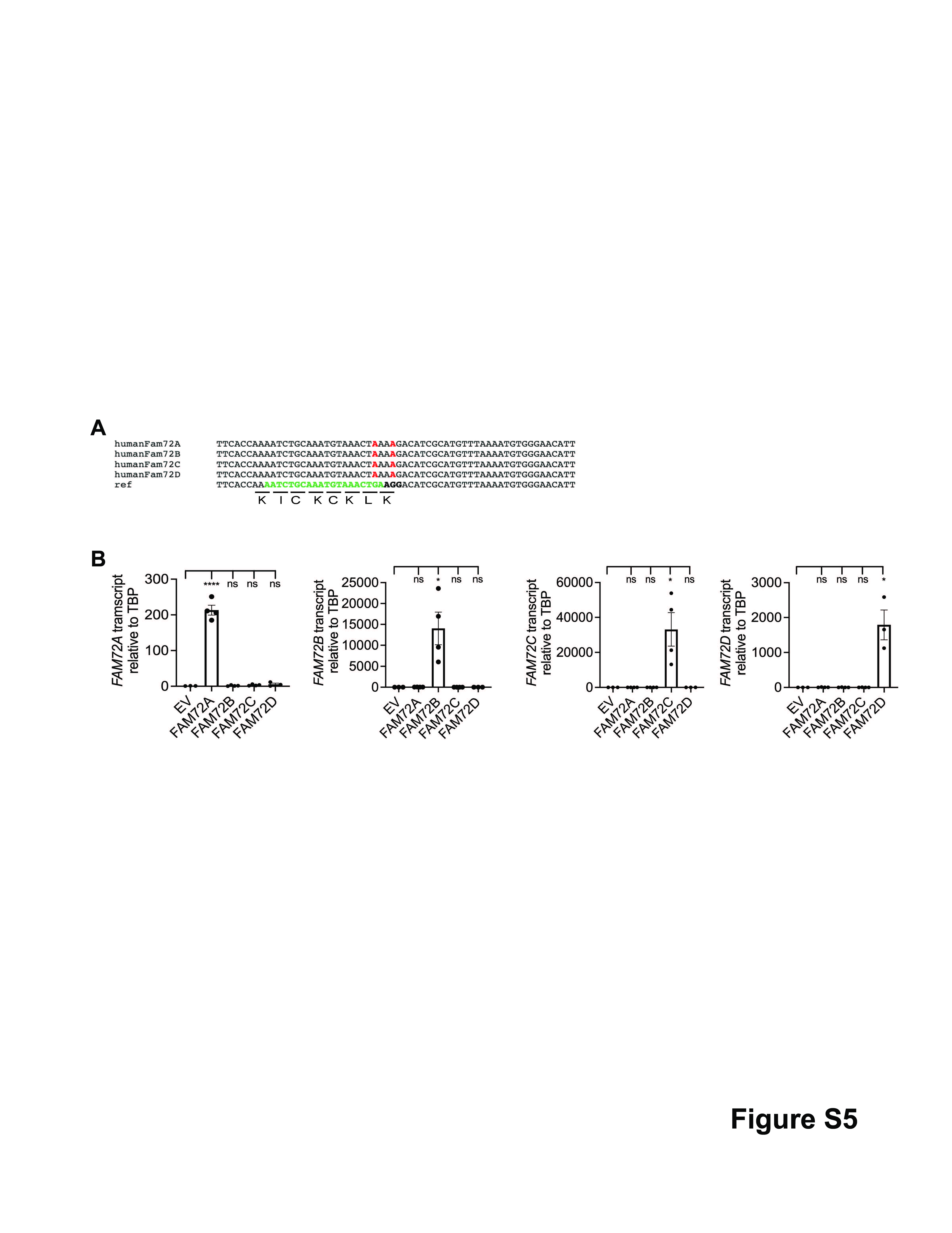
Figure S5. Expressing *FAM72* paralogues ectopically in wildtype and *FAM72*^-/-^ cells.** (**A**) synonymous mutations were introduced into *FAM72* exon2 guide RNA (gRNA) binding site and PAM sequence. gRNA sequence denoted in green, and introduced point mutations denoted in red. Bold letters highlight the PAM sequence. **(B)** *FAM72* transcript level in *FAM72*^-/-^ HCT116 clone #1 transduced with lentiviruses expressing *FAM72A, B, C, or D* genes. *FAM72* transcript level was normalized to the housekeeping gene *TBP* and compared to the *FAM72* expression level present in pMX-PIE empty vector (EV) control. Two-tailed unpaired Student’s t-test was used for statistically analysis. *, P < 0.05; **, P < 0.01; ***, P < 0.001 ****; *P < 0.0001*.
